# The genome sequence of the Red Chestnut moth, *Cerastis rubricosa *(Schiffermüller, 1775)

**DOI:** 10.12688/wellcomeopenres.20834.1

**Published:** 2024-02-19

**Authors:** Douglas Boyes, Peter W.H. Holland, University of Oxford and Wytham Woods Genome Acquisition Lab, Darwin Tree of Life Barcoding collective, Wellcome Sanger Institute Tree of Life Management, Samples and Laboratory team, Wellcome Sanger Institute Scientific Operations: Sequencing Operations, Wellcome Sanger Institute Tree of Life Core Informatics team, Tree of Life Core Informatics collective, Darwin Tree of Life Consortium

**Affiliations:** 1 UK Centre for Ecology & Hydrology, Wallingford, England, UK; 2 University of Oxford, Oxford, England, UK

**Keywords:** Cerastis rubricosa, Red Chestnut moth, genome sequence, chromosomal, Lepidoptera

## Abstract

We present a genome assembly from an individual male *Cerastis rubricosa* (the Red Chestnut moth; Arthropoda; Insecta; Lepidoptera; Noctuidae). The genome sequence is 678.7 megabases in span. Most of the assembly is scaffolded into 31 chromosomal pseudomolecules, including the Z sex chromosome. The mitochondrial genome has also been assembled and is 15.39 kilobases in length. Gene annotation of this assembly on Ensembl identified 18,784 protein coding genes.

## Species taxonomy

Eukaryota; Opisthokonta; Metazoa; Eumetazoa; Bilateria; Protostomia; Ecdysozoa; Panarthropoda; Arthropoda; Mandibulata; Pancrustacea; Hexapoda; Insecta; Dicondylia; Pterygota; Neoptera; Endopterygota; Amphiesmenoptera; Lepidoptera; Glossata; Neolepidoptera; Heteroneura; Ditrysia; Obtectomera; Noctuoidea; Noctuidae; Noctuinae; Noctuini; *Cerastis*; *Cerastis rubricosa* (Schiffermüller, 1775) (NCBI:txid988089).

## Background

The Red Chestnut *Cerastis rubricosa* is a reddish-brown spring-flying noctuid moth recorded widely across central and northern Europe, with additional scattered records from Russia, Ukraine, China and Hokkaido, Japan ( GBIF Secretariat, 2023). In Britain, the species is found from the north of Scotland to the south coast of England, through south and west Wales and across much of Northern Ireland, but has declined greatly in abundance over the past 50 years ( Randle *et al.*, 2019). In Ireland, *C. rubricosa* is mainly found in coastal regions and can be locally common ( MothsIreland, 2023).

In Europe the moth has one generation per year, with adults recorded from March to May. The polyphagous larvae feed in summer on a wide range of low-growing herbaceous plants including dock *Rumex* sp., dandelion *Taraxacum officinale*, groundsel *Senecio vulgaris* and orchids ( Sletvold *et al.*, 2015; South, 1961). The larvae are highly mobile and can move between plants to select their preferred food source ( Sletvold *et al.*, 2015). In a study of herbivory on the Fragrant orchid *Gymnadenia conopsea* in Norway, larvae of *C. rubricosa* were found to feed on orchid flowers before switching to feed on the leaves and stem, sometimes eating all parts of the plant above ground ( Sletvold *et al.*, 2015).

A genome sequence of the Red Chestnut *Cerastis rubricosa* was determined as part of the Darwin Tree of Life project. The genome sequence will facilitate research into adaptations to polyphagy and will contribute to the growing set of resources for studying molecular evolution in the Lepidoptera.

## Genome sequence report

**Figure 1.**
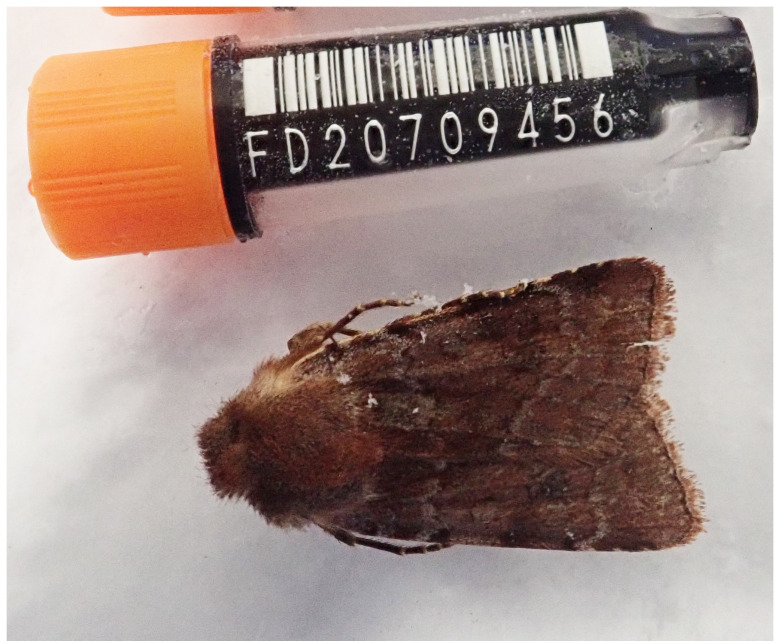
Photograph of the *Cerastis rubricosa* (ilCerRubr1) specimen used for genome sequencing.

The genome was sequenced from one male *Cerastis rubricosa* ( Figure 1) collected from Wytham Woods, Oxfordshire, UK (51.77, –1.34). A total of 34-fold coverage in Pacific Biosciences single-molecule HiFi long reads was generated. Primary assembly contigs were scaffolded with chromosome conformation Hi-C data. Manual assembly curation corrected 14 missing joins or mis-joins and removed 8 haplotypic duplications, reducing the assembly length by 1.46% and the scaffold number by 2.70%, and decreasing the scaffold N50 by 1.80%.

**Figure 2.**
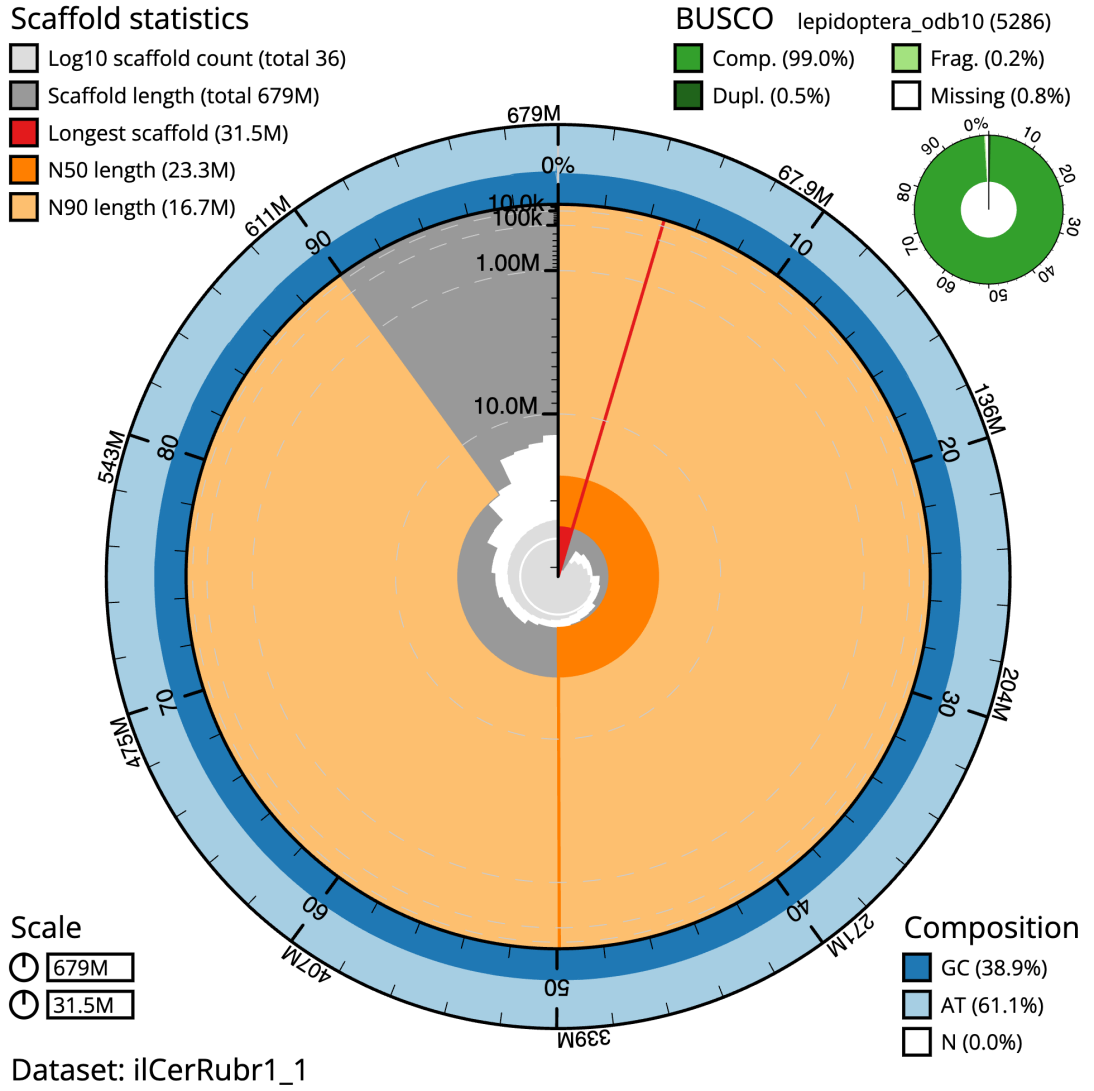
Genome assembly of *Cerastis rubricosa*, ilCerRubr1.1: metrics. The BlobToolKit Snailplot shows N50 metrics and BUSCO gene completeness. The main plot is divided into 1,000 size-ordered bins around the circumference with each bin representing 0.1% of the 678,709,332 bp assembly. The distribution of scaffold lengths is shown in dark grey with the plot radius scaled to the longest scaffold present in the assembly (31,522,147 bp, shown in red). Orange and pale-orange arcs show the N50 and N90 scaffold lengths (23,337,995 and 16,738,118 bp), respectively. The pale grey spiral shows the cumulative scaffold count on a log scale with white scale lines showing successive orders of magnitude. The blue and pale-blue area around the outside of the plot shows the distribution of GC, AT and N percentages in the same bins as the inner plot. A summary of complete, fragmented, duplicated and missing BUSCO genes in the lepidoptera_odb10 set is shown in the top right. An interactive version of this figure is available at https://blobtoolkit.genomehubs.org/view/ilCerRubr1_1/dataset/ilCerRubr1_1/snail.

**Figure 3.**
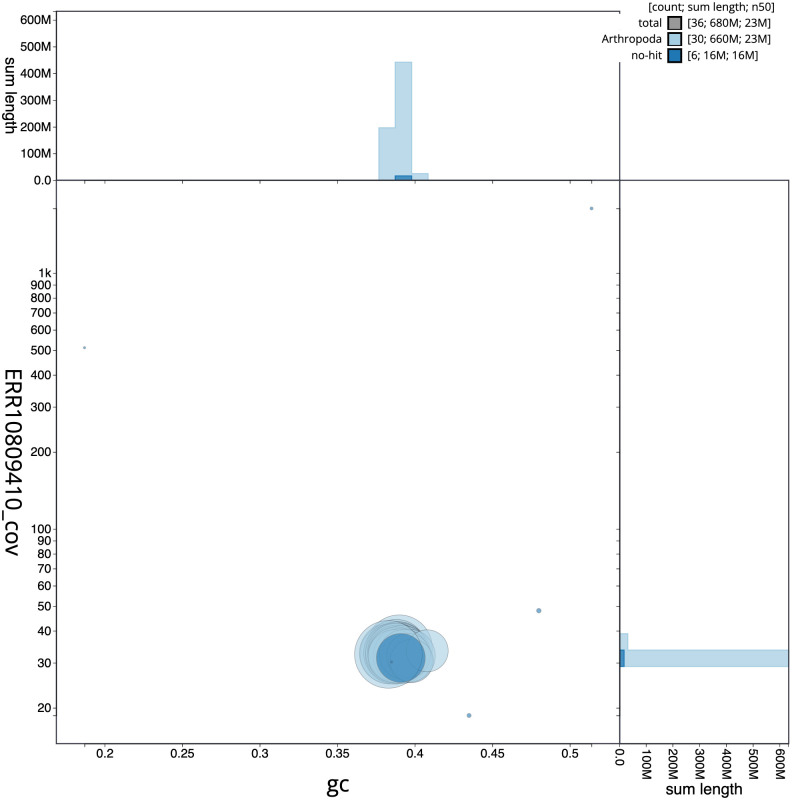
Genome assembly of *Cerastis rubricosa*, ilCerRubr1.1: BlobToolKit GC-coverage plot. Scaffolds are coloured by phylum. Circles are sized in proportion to scaffold length. Histograms show the distribution of scaffold length sum along each axis. An interactive version of this figure is available at https://blobtoolkit.genomehubs.org/view/ilCerRubr1_1/dataset/ilCerRubr1_1/blob.

**Figure 4.**
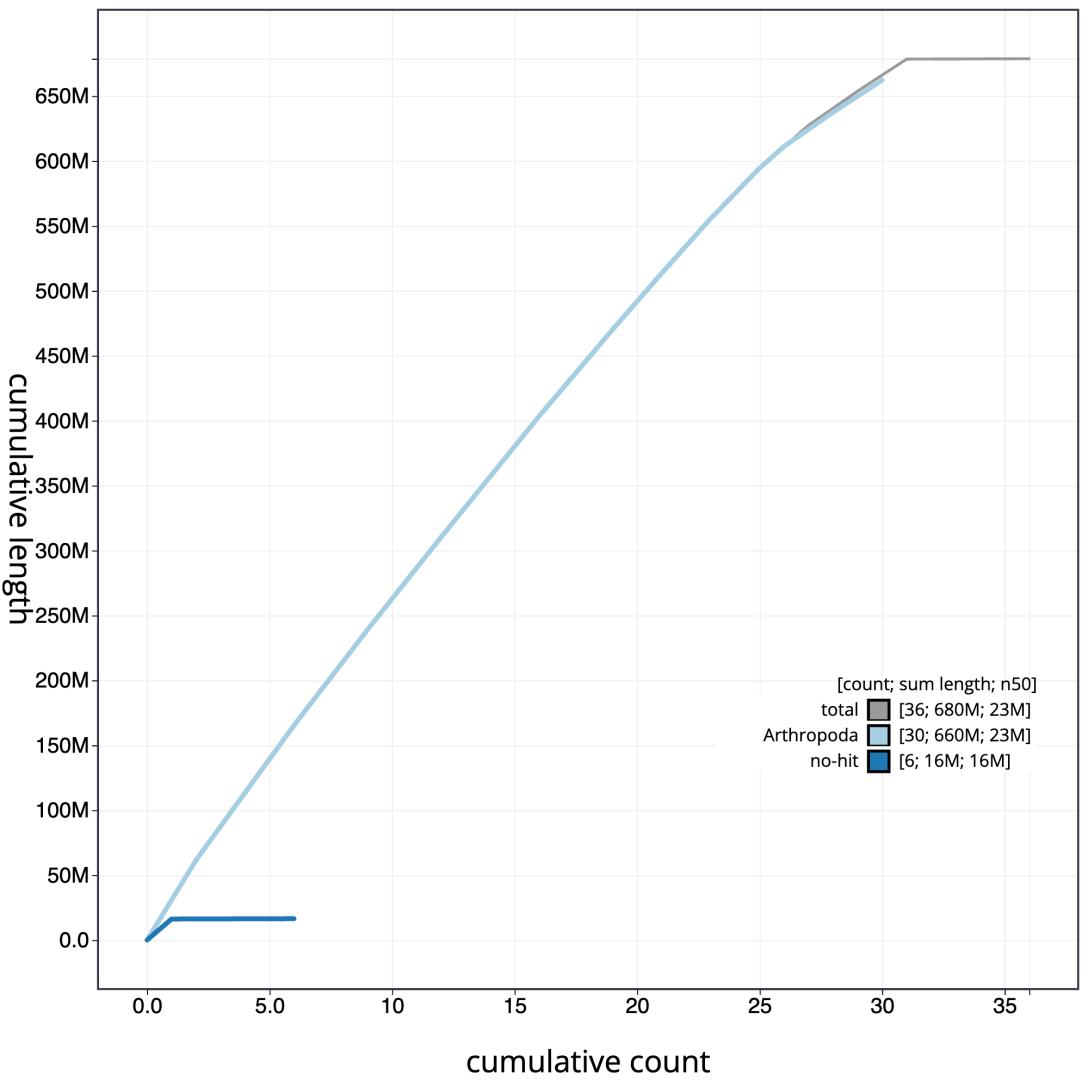
Genome assembly of *Cerastis rubricosa*, ilCerRubr1.1: BlobToolKit cumulative sequence plot. The grey line shows cumulative length for all scaffolds. Coloured lines show cumulative lengths of scaffolds assigned to each phylum using the buscogenes taxrule. An interactive version of this figure is available at https://blobtoolkit.genomehubs.org/view/ilCerRubr1_1/dataset/ilCerRubr1_1/cumulative.

**Figure 5.**
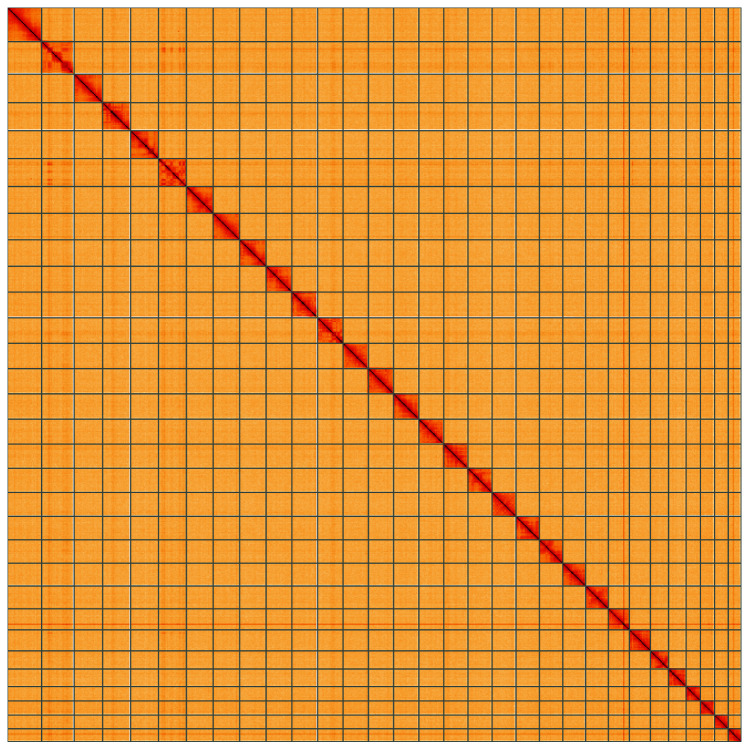
Genome assembly of *Cerastis rubricosa*, ilCerRubr1.1: Hi-C contact map of the ilCerRubr1.1 assembly, visualised using HiGlass. Chromosomes are shown in order of size from left to right and top to bottom. An interactive version of this figure may be viewed at https://genome-note-higlass.tol.sanger.ac.uk/l/?d=aghxpM00RxKIO-eZme_jEA.

The final assembly has a total length of 678.7 Mb in 35 sequence scaffolds with a scaffold N50 of 23.3 Mb ( Table 1). The snailplot in Figure 2 provides a summary of the assembly statistics, while the distribution of assembly scaffolds on GC proportion and coverage is shown in Figure 3. The cumulative assembly plot in Figure 4 shows curves for subsets of scaffolds assigned to different phyla. Most (99.96%) of the assembly sequence was assigned to 31 chromosomal-level scaffolds, representing 30 autosomes and the Z sex chromosome. Chromosome-scale scaffolds confirmed by the Hi-C data are named in order of size ( Figure 5; Table 2). The Z chromosome identified based on synteny with *Diarsia rubi* (GCA_932274075.1) ( Boyes *et al.*, 2023). While not fully phased, the assembly deposited is of one haplotype. Contigs corresponding to the second haplotype have also been deposited. The mitochondrial genome was also assembled and can be found as a contig within the multifasta file of the genome submission.

**Table 1.** Genome data for *Cerastis rubricosa*, ilCerRubr1.1.

Project accession data		
Assembly identifier	ilCerRubr1.1	
Species	*Cerastis rubricosa*	
Specimen	ilCerRubr1	
NCBI taxonomy ID	988089	
BioProject	PRJEB59288	
BioSample ID	SAMEA10107029	
Isolate information	ilCerRubr1, male: abdomen (DNA sequencing), head (Hi-C sequencing)	
Assembly metrics *		*Benchmark*
Consensus quality (QV)	68.7	*≥ 50*
*k*-mer completeness	100.0%	*≥ 95%*
BUSCO **	C:99.0%[S:98.5%,D:0.5%], F:0.2%,M:0.8%,n:5,286	*C ≥ 95%*
Percentage of assembly mapped to chromosomes	99.96%	*≥ 95%*
Sex chromosomes	Z	*localised * *homologous * *pairs*
Organelles	Mitochondrial genome: 15.39 kb	*complete * *single alleles*
Raw data accessions		
PacificBiosciences SEQUEL II	ERR10809410	
Hi-C Illumina	ERR10818316	
Genome assembly		
Assembly accession	GCA_949152445.1	
*Accession of alternate haplotype*	GCA_949152405.1	
Span (Mb)	678.7	
Number of contigs	107	
Contig N50 length (Mb)	11.0	
Number of scaffolds	35	
Scaffold N50 length (Mb)	23.3	
Longest scaffold (Mb)	31.52	
Genome annotation		
Number of protein-coding genes	18,784	
Number of gene transcripts	18,993	

* Assembly metric benchmarks are adapted from column VGP-2020 of “Table 1: Proposed standards and metrics for defining genome assembly quality” from ( Rhie *et al.*, 2021). ** BUSCO scores based on the lepidoptera_odb10 BUSCO set using version 5.3.2. C = complete [S = single copy, D = duplicated], F = fragmented, M = missing, n = number of orthologues in comparison. A full set of BUSCO scores is available at https://blobtoolkit.genomehubs.org/view/ilCerRubr1_1/dataset/ilCerRubr1_1/busco.

**Table 2.** Chromosomal pseudomolecules in the genome assembly of *Cerastis rubricosa*, ilCerRubr1.

INSDC accession	Chromosome	Length (Mb)	GC%
OX424524.1	1	30.09	39.0
OX424525.1	2	26.31	39.0
OX424526.1	3	25.88	39.0
OX424527.1	4	25.8	38.5
OX424528.1	5	25.8	39.0
OX424529.1	6	24.74	38.5
OX424530.1	7	24.49	39.0
OX424531.1	8	24.47	38.5
OX424532.1	9	23.86	38.5
OX424533.1	10	23.76	39.0
OX424534.1	11	23.57	39.0
OX424535.1	12	23.39	39.0
OX424536.1	13	23.34	39.0
OX424537.1	14	23.33	38.5
OX424538.1	15	23.07	38.5
OX424539.1	16	22.37	39.0
OX424540.1	17	22.32	39.0
OX424541.1	18	22.11	39.0
OX424542.1	19	21.66	39.0
OX424543.1	20	21.62	39.0
OX424544.1	21	21.07	39.0
OX424545.1	22	21.05	39.0
OX424546.1	23	19.48	39.5
OX424547.1	24	19.41	38.5
OX424548.1	25	16.74	39.5
OX424549.1	26	16.24	39.0
OX424550.1	27	13.25	39.5
OX424551.1	28	13.04	39.5
OX424552.1	29	12.68	40.0
OX424553.1	30	11.99	41.0
OX424523.1	Z	31.52	38.5
OX424554.1	MT	0.02	19.0

The estimated Quality Value (QV) of the final assembly is 68.7 with *k*-mer completeness of 100.0%, and the assembly has a BUSCO v5.3.2 completeness of 99.0% (single = 98.5%, duplicated = 0.5%), using the lepidoptera_odb10 reference set ( *n* = 5,286).

Metadata for specimens, barcode results, spectra estimates, sequencing runs, contaminants and pre-curation assembly statistics are given at https://links.tol.sanger.ac.uk/species/988089.

## Genome annotation report

The *Cerastis rubricosa* genome assembly (GCA_949152445.1) was annotated using the Ensembl rapid annotation pipeline ( Table 1; https://rapid.ensembl.org/Cerastis_rubricosa_GCA_949152445.1/Info/Index). The resulting annotation includes 18,993 transcribed mRNAs from 18,784 protein-coding genes.

## Methods

### Sample acquisition and nucleic acid extraction

A male *Cerastis rubricosa* (specimen ID Ox001104, ToLID ilCerRubr1) was collected from Wytham Woods, Oxfordshire (biological vice-county Berkshire), UK (latitude 51.77, longitude –1.34) on 2021-03-31 using a light trap. The specimen was collected and identified by Douglas Boyes (University of Oxford) and preserved on dry ice.

The workflow for high molecular weight (HMW) DNA extraction at the Wellcome Sanger Institute (WSI) includes a sequence of core procedures: sample preparation; sample homogenisation, DNA extraction, fragmentation, and clean-up. In sample preparation, the ilCerRubr1 sample was weighed and dissected on dry ice ( Jay *et al.*, 2023). For sample homogenisation, abdomen tissue was cryogenically disrupted using the Covaris cryoPREP ^®^ Automated Dry Pulverizer ( Narváez-Gómez *et al.*, 2023). HMW DNA was extracted using the Automated MagAttract v1 protocol ( Sheerin *et al.*, 2023). DNA was sheared into an average fragment size of 12–20 kb in a Megaruptor 3 system with speed setting 30 ( Todorovic *et al.*, 2023). Sheared DNA was purified by solid-phase reversible immobilisation ( Strickland *et al.*, 2023): in brief, the method employs a 1.8X ratio of AMPure PB beads to sample to eliminate shorter fragments and concentrate the DNA. The concentration of the sheared and purified DNA was assessed using a Nanodrop spectrophotometer and Qubit Fluorometer and Qubit dsDNA High Sensitivity Assay kit. Fragment size distribution was evaluated by running the sample on the FemtoPulse system.

Protocols developed by the WSI Tree of Life laboratory are publicly available on protocols.io ( Denton *et al.*, 2023).

### Sequencing

Pacific Biosciences HiFi circular consensus DNA sequencing libraries were constructed according to the manufacturers’ instructions. DNA sequencing was performed by the Scientific Operations core at the WSI on a Pacific Biosciences SEQUEL II (HiFi) instrument. Hi-C data were also generated from head tissue of ilCerRubr1 using the Arima2 kit and sequenced on the Illumina NovaSeq 6000 instrument.

### Genome assembly, curation and evaluation

Assembly was carried out with Hifiasm ( Cheng *et al.*, 2021) and haplotypic duplication was identified and removed with purge_dups ( Guan *et al.*, 2020). The assembly was then scaffolded with Hi-C data ( Rao *et al.*, 2014) using YaHS ( Zhou *et al.*, 2023). The assembly was checked for contamination and corrected as described previously ( Howe *et al.*, 2021). Manual curation was performed using HiGlass ( Kerpedjiev *et al.*, 2018) and Pretext ( Harry, 2022). The mitochondrial genome was assembled using MitoHiFi ( Uliano-Silva *et al.*, 2023), which runs MitoFinder ( Allio *et al.*, 2020) or MITOS ( Bernt *et al.*, 2013) and uses these annotations to select the final mitochondrial contig and to ensure the general quality of the sequence.

A Hi-C map for the final assembly was produced using bwa-mem2 ( Vasimuddin *et al.*, 2019) in the Cooler file format ( Abdennur & Mirny, 2020). To assess the assembly metrics, the *k*-mer completeness and QV consensus quality values were calculated in Merqury ( Rhie *et al.*, 2020). This work was done using Nextflow ( Di Tommaso *et al.*, 2017) DSL2 pipelines “sanger-tol/readmapping” ( Surana *et al.*, 2023a) and “sanger-tol/genomenote” ( Surana *et al.*, 2023b). The genome was analysed within the BlobToolKit environment ( Challis *et al.*, 2020) and BUSCO scores ( Manni *et al.*, 2021; Simão *et al.*, 2015) were calculated.

Table 3 contains a list of relevant software tool versions and sources.

**Table 3.** Software tools: versions and sources.

Software tool	Version	Source
BlobToolKit	4.2.1	https://github.com/blobtoolkit/blobtoolkit
BUSCO	5.3.2	https://gitlab.com/ezlab/busco
Hifiasm	0.16.1-r375	https://github.com/chhylp123/hifiasm
HiGlass	1.11.6	https://github.com/higlass/higlass
Merqury	MerquryFK	https://github.com/thegenemyers/MERQURY.FK
MitoHiFi	2	https://github.com/marcelauliano/MitoHiFi
PretextView	0.2	https://github.com/wtsi-hpag/PretextView
purge_dups	1.2.3	https://github.com/dfguan/purge_dups
sanger-tol/ genomenote	v1.0	https://github.com/sanger-tol/genomenote
sanger-tol/ readmapping	1.1.0	https://github.com/sanger-tol/readmapping/tree/1.1.0
YaHS	1.2a	https://github.com/c-zhou/yahs

### Genome annotation

The BRAKER2 pipeline ( Brůna *et al.*, 2021) was used in the default protein mode to generate annotation for the *Cerastis rubricosa* assembly (GCA_949152445.1) in Ensembl Rapid Release.

### Wellcome Sanger Institute – Legal and Governance

The materials that have contributed to this genome note have been supplied by a Darwin Tree of Life Partner. The submission of materials by a Darwin Tree of Life Partner is subject to the **‘Darwin Tree of Life Project Sampling Code of Practice’**, which can be found in full on the Darwin Tree of Life website here. By agreeing with and signing up to the Sampling Code of Practice, the Darwin Tree of Life Partner agrees they will meet the legal and ethical requirements and standards set out within this document in respect of all samples acquired for, and supplied to, the Darwin Tree of Life Project. 

Further, the Wellcome Sanger Institute employs a process whereby due diligence is carried out proportionate to the nature of the materials themselves, and the circumstances under which they have been/are to be collected and provided for use. The purpose of this is to address and mitigate any potential legal and/or ethical implications of receipt and use of the materials as part of the research project, and to ensure that in doing so we align with best practice wherever possible. The overarching areas of consideration are:

• Ethical review of provenance and sourcing of the material

• Legality of collection, transfer and use (national and international) 

Each transfer of samples is further undertaken according to a Research Collaboration Agreement or Material Transfer Agreement entered into by the Darwin Tree of Life Partner, Genome Research Limited (operating as the Wellcome Sanger Institute), and in some circumstances other Darwin Tree of Life collaborators.

## Data Availability

European Nucleotide Archive: *Cerastis rubricosa* (red chestnut). Accession number PRJEB59288; https://identifiers.org/ena.embl/PRJEB59288 ( Wellcome Sanger Institute, 2023). The genome sequence is released openly for reuse. The *Cerastis rubricosa* genome sequencing initiative is part of the Darwin Tree of Life (DToL) project. All raw sequence data and the assembly have been deposited in INSDC databases. Raw data and assembly accession identifiers are reported in Table 1.

## Author information

Members of the University of Oxford and Wytham Woods Genome Acquisition Lab are listed here: https://doi.org/10.5281/zenodo.7125292.

Members of the Darwin Tree of Life Barcoding collective are listed here: https://doi.org/10.5281/zenodo.4893703.

Members of the Wellcome Sanger Institute Tree of Life Management, Samples and Laboratory team are listed here: https://doi.org/10.5281/zenodo.10066175.

Members of Wellcome Sanger Institute Scientific Operations: Sequencing Operations are listed here: https://doi.org/10.5281/zenodo.10043364.

Members of the Wellcome Sanger Institute Tree of Life Core Informatics team are listed here: https://doi.org/10.5281/zenodo.10066637.

Members of the Tree of Life Core Informatics collective are listed here: https://doi.org/10.5281/zenodo.5013541.

Members of the Darwin Tree of Life Consortium are listed here: https://doi.org/10.5281/zenodo.4783558.
