# Supplementary material for: The genome sequence of the Red Chestnut moth, Cerastis rubricosa (Schiffermüller, 1775)
Source: Wellcome Open Res. 2024 Feb 19;9:88. [Version 1] doi: 10.12688/wellcomeopenres.20834.1 (PMC11406127; doi:10.12688/wellcomeopenres.20834.1)
Supplement: Peer review history

# Reviewer report: Reviewer response for version 1

**DOI:** 10.21956/wellcomeopenres.23053.r90349

**Published:** 2024-09-16

**Authors:** Sarah Inwood

**Affiliations:** 1 University of Otago, Dunedin, Otago, New Zealand

The authors sequenced and assembled a chromosomal scale genome assembly for the Red Chestnut moth *Cerastis rubricosa*. This assembly used HiFi long reads and Hi-C data, resulting in a high-quality assembly, alongside a mitochondrial genome assembly.

**Minor comments**:

1. Are the BUSCO results reported on the genome itself or the gene annotations, and how much variation was there between both BUSCO results?
2. The authors state the use of MitoHifi, which can run MitoFinder OR MITOS, but do not state which tool resulted in their deposited mitochondrial genome assembly.
3. As stated by the other reviewer, stating the chromosomal sex determination method and variation in sequencing depth of the Z chromosome compared to others would be beneficial.

Are sufficient details of methods and materials provided to allow replication by others?

Yes

Is the rationale for creating the dataset(s) clearly described?

Yes

Are the datasets clearly presented in a useable and accessible format?

Yes

Are the protocols appropriate and is the work technically sound?

Yes

Reviewer Expertise:

Genomics, bioinformatics, host-parasite interactions, parasitoid wasps, insects, viruses, biocontrol

I confirm that I have read this submission and believe that I have an appropriate level of expertise to confirm that it is of an acceptable scientific standard.

# Reviewer report: Reviewer response for version 1

**DOI:** 10.21956/wellcomeopenres.23053.r90348

**Published:** 2024-08-06

**Authors:** Andrew J. Veale

**Affiliations:** 1 Manaaki Whenua Landcare Research, Lincoln, New Zealand

The methods appear fine.  Stating the chromosome sex determination method of this species would be useful - I assume it is males are ZZ and females ZO?  Was the depth the same for the Z as for other chromosomes?  Was species ID confirmed using the mitochondrial sequence?

 Other than that nothing further required.

Are sufficient details of methods and materials provided to allow replication by others?

Yes

Is the rationale for creating the dataset(s) clearly described?

Yes

Are the datasets clearly presented in a useable and accessible format?

Yes

Are the protocols appropriate and is the work technically sound?

Yes

Reviewer Expertise:

Wildlife ecology and genomics

I confirm that I have read this submission and believe that I have an appropriate level of expertise to confirm that it is of an acceptable scientific standard.
